# High genetic diversity among and within bitter manioc varieties cultivated in different soil types in Central Amazonia

**DOI:** 10.1590/1678-4685-GMB-2016-0046

**Published:** 2017-04-10

**Authors:** Alessandro Alves-Pereira, Nivaldo Peroni, Marcelo Mattos Cavallari, Maristerra R. Lemes, Maria Imaculada Zucchi, Charles R. Clement

**Affiliations:** 1 Departamento de Genética, Escola Superior de Agricultura “Luiz de Queiróz”, Universidade de São Paulo, Piracicaba, SP, Brazil; 2 Laboratório de Evolução Aplicada, Universidade Federal do Amazonas, Manaus, AM, Brazil; 3 Universidade Federal de Santa Catarina, Departamento de Ecologia e Zoologia, Florianópolis, SC, Brazil; 4 Empresa Brasileira de Pesquisa Agropecuária, EMBRAPA COCAIS, São Luis, MA, Brazil; 5 Instituto Nacional de Pesquisas da Amazônia, Manaus, AM, Brazil; 6 Laboratório de Genética e Biologia Reprodutiva de Plantas, Instituto Nacional de Pesquisas da Amazônia, Manaus, AM, Brazil; 7 Agência Paulista de Tecnologia dos Agronegócios, Pólo Regional Centro-Sul, Piracicaba, SP, Brazil

**Keywords:** Manihot esculenta, microsatellites, Amazonian dark earths, floodplain, Oxisols

## Abstract

Although manioc is well adapted to nutrient-poor Oxisols of Amazonia, ethnobotanical observations show that bitter manioc is also frequently cultivated in the highly fertile soils of the floodplains and Amazonian dark earths (ADE) along the middle Madeira River. Because different sets of varieties are grown in each soil type, and there are agronomic similarities between ADE and floodplain varieties, it was hypothesized that varieties grown in ADE and floodplain were more closely related to each other than either is to varieties grown in Oxisols. We tested this hypothesis evaluating the intra-varietal genetic diversity and the genetic relationships among manioc varieties commonly cultivated in Oxisols, ADE and floodplain soils. Genetic results did not agree with ethnobotanical expectation, since the relationships between varieties were variable and most individuals of varieties with the same vernacular name, but grown in ADE and floodplain, were distinct. Although the same vernacular name could not always be associated with genetic similarities, there is still a great amount of variation among the varieties. Many ecological and genetic processes may explain the high genetic diversity and differentiation found for bitter manioc varieties, but all contribute to the maintenance and amplification of genetic diversity within the manioc in Central Amazonia.

## Introduction

Manioc (*Manihot esculenta* Crantz ssp. *esculenta*) was domesticated in southwestern Amazonia at least 7,000 years ago (Allem, 1994; Olsen and Schaal, 1999) and today is cultivated in all tropical countries (Lebot, 2009). It is grown for its tuberous starchy roots, which are the primary source of carbohydrates for more than 800 million people (Lebot, 2009). Cultivated manioc is classified into two major groups of varieties. The “sweet” varieties have low amounts of cyanogenic glycosides (< 100 ppm fresh weight), while “bitter” varieties have high amounts of these toxic substances (> 100 ppm fresh weight) (McKey *et al*., 2010a). The discrimination of these two groups is recognized by traditional farmers (Rival and McKey, 2008), and supported by molecular markers (Mühlen *et al*., 2000; Peroni *et al*., 2007).

There is strong selection for manioc varieties with high toxicity, especially in Central Amazonia where the majority of varieties are bitter (McKey and Beckerman, 1993). Bitter manioc is most frequently grown on the nutrient-poor Oxisols of non-flooded upland plateaus (Fraser and Clement, 2008). However, manioc is also cultivated in the highly fertile Fluvent Entisols found in the floodplains of Amazonian white-water rivers (Gutjahr, 2000). Bitter manioc is also a widespread crop in the highly fertile Amazonian dark earths (ADE) used by communities of smallholder farmers along the middle Madeira River (Fraser, 2010). ADE are anthropogenic soils, located on upland plateaus, associated with Amerindian settlements from the pre-Columbian period (Glaser and Birk, 2012), and are as fertile as the floodplain soils. Despite the importance of manioc cultivation on high-fertility soils, all previously published research on manioc genetic diversity in Amazonia was undertaken in environments of low soil fertility.

In Amazonia, manioc is generally cultivated in low-input swidden-fallow systems of traditional farmers (Emperaire, 2005), where secondary vegetation is cleared and burnt, and the swiddens are used for cultivation and then fallowed. Secondary vegetation is left to grow for a variable period before the area is again used for cultivation (Elias *et al*., 2000). Although the cultivation of manioc is based exclusively on clonal propagation, sexual reproduction may be a very common spontaneous event as volunteer seedlings sprout after the burn and grow at the same time as plants that were vegetatively propagated (Pujol *et al*., 2007). Farmers may let the seedlings grow and by harvesting time they may decide to propagate some of these plants vegetatively (Rival and McKey, 2008). If so, farmers may either incorporate them into an existing variety or assign a new varietal name (Rival and McKey, 2008). The process of incorporation of seedlings is essential to maintain genetic diversity within the crop (Pujol *et al*., 2007; Duputié *et al*., 2009) and results in polyclonal varieties.

Based on ethnobotanical observation, Fraser (2010) suggested that traditional farmers along the middle Madeira River manage distinct sets of varieties according to their suitability for the different environments of cultivation, which are characterized principally by soil type and by the occurrence, or not, of annual flooding (ADE and Oxisols on the uplands *vs*. Entisols in the floodplain). Soil type is classified by traditional farmers as “weak” (soils that are more intensively cultivated, with short cropping cycles and shorter fallowing periods, such as ADE soils) and “strong” (soils that are more extensively cultivated, with short cycles of cultivation and longer fallowing periods, such as Oxisols). These authors observed that local varieties of bitter manioc are termed “weak” (low starch, fast maturing) or “strong” (high starch, slow maturing) according to their suitability for cultivation in “weak” or “strong” soils, respectively. In addition, due to the seasonality of flood events, bitter manioc varieties cultivated in the floodplain mature quickly (like “weak” varieties). For these reasons, the authors hypothesized that the “weak” varieties have origins traceable to the floodplain soils, and thus one would expect that the varieties grown in ADE are genetically related to the varieties grown in the floodplain. A few interviews with farmers also supported this hypothesis (Fraser, 2010).

Fraser (2010) also observed that the classification of the soils may change according to their intensity of use, and farmers may select a new set of varieties more suitable for the soil type (Fraser and Clement, 2008). This variation in the perception of the suitability of varieties for different soil types may cause, for example, farmers to grow low starch fast maturing “weak” varieties on “strong” soils (Oxisols) that are more intensively cultivated. Therefore, gene flow among varieties adapted to different soil types is probable through sexual reproduction and incorporation of seedlings.

Despite the existence of distinct sets of varieties preferentially grown in each soil type, farmers along the middle Madeira River may still cultivate varieties with the same vernacular name in different environments than the most suitable one (Fraser *et al*., 2012). Therefore, the cultivation system of this region is an ideal scenario to evaluate the genetic similarities and relationships among bitter manioc varieties in light of these ethnobotanical expectations. The objective of this study was to test a hypothesis raised by ethnobotanical observations that manioc varieties from ADE and the floodplain are genetically more closely related to each other, based on the assessment of genetic diversity using molecular tools. We evaluated the genetic diversity and structure among 14 of the most common varieties grown in different soil types in smallholder farming communities along the middle Madeira River region using ten microsatellite loci. The patterns of genetic diversity are discussed with the aim of integrating the accumulated knowledge on genetic diversity and ethnobotany of the crop.

## Material and Methods

### Sampling sites and permission

**Figure 1:**
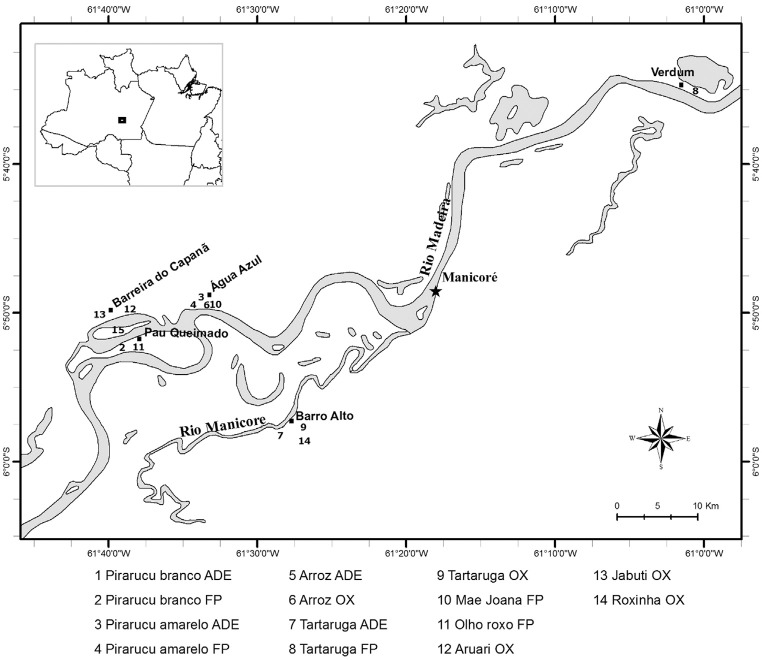
Map showing the communities of smallholder farmers in the municipality of Manicoré, along the middle Madeira River, Amazonas, Brazil, where manioc varieties were sampled. The numbers correspond to the varieties' names and the soil types in which they were grown. Soil types are coded as ADE (Amazonian dark earths), FP (floodplain) and OX (Oxisols).

The manioc varieties analyzed in this study are amongst the most frequently grown in different soil types found in Manicoré (5°18′S; 61°18′W), an essentially agricultural municipality located in Amazonas state, Brazil, along the middle Madeira River (Fraser, 2010). The manioc varieties were sampled in the riverside non-indigenous communities of Água Azul, Barreira do Capanã, Barro Alto, Pau Queimado and Verdum (Figure 1), which are composed of smallholder farmers who grow manioc in traditional swidden-fallow systems in upland ADE and Oxisols, as well as in floodplain soils. At each community, the project goals were explained to the farmers, in order to obtain prior informed consent. This work met Resolution 21 requirements for basic research and was exempted from authorization by Brazil's Council for Genetic Patrimony (CGEN in the Brazilian acronym), which was consulted before field work. Our study was authorized by the *Instituto Nacional de Pesquisas da Amazônia*'s Committee for Research Ethics (Permit number 235/09) and our collecting was registered in the System for Authorization and Information on Biodiversity, coordinated by the Chico Mendes Institute for Biodiversity of the Ministry of the Environment (number of register: 10020-5).

A total of 14 manioc varieties (with nine different vernacular names) were sampled: four in ADE, five in the floodplain and five in Oxisols (Table 1). Leaves were collected, dried in silica gel, and stored at -20 °C until DNA extraction.

**Table 1:** Manioc varieties sampled in different soils of different communities of smallholder farmers in the municipality of Manicoré, along the middle Madeira River, Amazonas, Brazil. N = number of individuals sampled.

Vernacular name	Soil	Variety Acronym	N	Community
*Pirarucu Branco*	ADE	PB ADE	20	Barreira do Capanã
	Floodplain	PB FP	20	Pau Queimado
*Pirarucu Amarelo*	ADE	PA ADE	30	Água Azul
	Floodplain	PA FP	30	Água Azul
*Arroz*	ADE	AR ADE	20	Barreira do Capanã
	Oxisol	AR OX	30	Água Azul
*Tartaruga*	ADE	TA ADE	30	Barro Alto
	Floodplain	TA FP	30	Verdum
	Oxisol	TA OX	30	Barro Alto
*Mãe Joana*	Floodplain	MJ FP	30	Água Azul
*Olho Roxo*	Floodplain	OR FP	30	Pau Queimado
*Aruari*	Oxisol	AU OX	30	Barreira do Capanã
*Jabuti*	Oxisol	JA OX	30	Barreira do Capanã
*Roxinha*	Oxisol	RO OX	30	Barro Alto

### DNA extraction and microsatellite genotyping

Genomic DNA was extracted from 50 mg of powdered leaf tissue using the CTAB 2% method described by Doyle and Doyle (1987) with a minor modification (the reagent 2-mercaptethanol was not used). DNA was quantified by comparison with known concentrations of standard DNA (lambda DNA, Fermentas, Carlsbad, USA) in electrophoresis agarose gels (0.9% w/v) stained with GelRed (Biotium Inc., San Francisco, USA).

Seven microsatellite markers (GA21, GA126, GA131, GA134, GA136, GA140, GAGG5) developed by Chavarriaga-Aguirre *et al*. (1998), and three (SSRY13, SSRY89, SSRY164) developed by Mba *et al*. (2001) were used. The microsatellite markers developed by Chavarriaga-Aguirre *et al*. (1998) were isolated from the MCol22 accession of the manioc germplasm collection at the International Centre for Tropical Agriculture, Cali, Colombia (CIAT), with the aim of the mapping and characterizing the genetic variability of CIAT's core collection. The microsatellites described by Mba *et al*. (2001) were isolated from the improved manioc variety TMS 30572, developed at the International Institute of Tropical Agriculture, Ibadan, Nigeria, (IITA), as part of an effort to generate molecular markers to saturate a genetic map of manioc. The utility of these microsatellite markers for the assessment of genetic diversity of manioc has been demonstrated in previous studies (Bradbury *et al*., 2013; Mühlen *et al*., 2013). PCR assays were carried out in a final volume of 10 μL with 20 ng of genomic DNA, 1X buffer (Mg^2+^ free), 2.5 ng of BSA, 2.5 mM of MgCl_2_, 250 μM of each dNTP, 2.5 pmols of each forward and reverse primer, and 1 U *Taq* DNA polymerase (Fermentas). Amplifications were carried out in a Verirti thermocycler (Applied Biosystems, Inc, Foster City, USA) as follows: an initial denaturation step of 94 °C for 2 min followed by 30 cycles at 94 °C for 1 min; 56 °C for 1 min; 72 °C for 2 min, and a final step of extension at 72 °C for 25 min. The quality of the PCR products was checked by electrophoresis in agarose gels (2% w/v) stained with GelRed. Forward sequences of the microsatellite primers were labeled with fluorescence (either FAM, 6-HEX or NED), and genotyping was performed in multiplexed systems in a DNA sequencer ABI3130xl (Applied Biosystems). GeneScanTM -500 ROX^TM^- size standard (Applied Biosystems) was used for allele sizing. Data collection and analysis were performed using GeneMapper v.4.0 (Applied Biosystems). According to the manufacturer, since the size standard is added to each sample, the process of calling alleles is very precise. However, as a standard procedure, in the case of samples that did not present clear patterns for allele calling, genotyping was performed more than once.

### Data analysis

#### Intra-varietal genetic diversity

Genetic diversity parameters of total (*A*) and mean (*Ā*) number of alleles, observed (*H*_*O*_) and expected (*H*_*E*_) heterozygosities, number of private alleles (*Ap*) and the inbreeding coefficient (*f*) were estimated with GenAlEx v.6.5 (Peakall and Smouse, 2012) for each locus and variety, and for the groups of varieties of each soil type. Significance of *f* was determined with Genetix v.4.03 (Belkhir *et al*., 2002), based on 10,000 bootstrap replicates. The recognition of identical and distinct multilocus genotypes (MLGs) was performed with GenClone v.2.0 (Arnaud-Haond and Belkhir, 2007) in order to investigate the presence of common MLGs among different varieties. Individuals with missing data were excluded from this analysis.

#### Genetic structure

Two methods were used to investigate the genetic structure and relationships among varieties. First we used discriminant analysis of principal components (DAPC, Jombart *et al*., 2010), a multivariate analysis with no underlying model run with the adegenet R package (Jombart and Ahmed, 2011). Previous to the analysis we used the *K*-means clustering algorithm, also implemented in adegenet, to set *K* = 11 as the prior grouping criterion (Figure S1). Based on these 11 clusters we ran DAPC retaining 10 principal components (corresponding to 98.73% of total variation). To access stability of group membership probabilities generated in DAPC, we used the a-score criterion also implemented in adegenet. We then compared the DAPC individuals' assignments to those obtained with TESS (Chen *et al*., 2007). TESS implements a Bayesian model based on Hidden Markov Random Field (HMRF), which models spatial dependencies at the cluster membership level of individuals being tested. The program searches for significant geographical discontinuities in allele frequencies determining population structure from individual multilocus genotypes sampled at different geographical locations. The interaction parameter ψ controls the importance given to spatial interactions, and when set to zero the HMRF model assumes a non-informative spatial prior, which corresponds to the no admixture, uncorrelated allele frequencies model implemented in Structure (Pritchard *et al*., 2000; Durand *et al*., 2009). TESS was run with the no admixture model, with ψ = zero for a period of 50,000 steps after a burn in period of 10,000. With these configurations we aimed to perform an analysis similar to what may be performed with Structure, but with the advantage of not having the constraints of the assumptions of Hardy-Weinberg equilibrium and minimization of linkage disequilibrium within clusters. Ten independent runs were performed for each value of the maximal number of clusters (*K*_max_), with *K*_max_ varying from two to 20. Selection of the *K*_max_ that best fit data was performed with the Deviance Information Criterion (DIC, Figure S1) in a way similar to the *ad hoc* Δ*K* criterion used for Structure results (Evanno *et al*., 2005).

The genetic differentiation among varieties was additionally investigated by estimating pairwise fixation indexes (*F*_*ST*_) using Arlequin v.3.5 (Excoffier and Lischer, 2010). Significance tests were carried out with 1,000 bootstrap replicates. Because varieties have a great number of individuals with identical MLGs, these estimates were calculated retaining only one individual per MLG found within each variety. Although the results of these analyses may be difficult to discuss in terms of genetic structuring, we aimed to evaluate allelic composition differentiation across varieties. To evaluate the distribution of genetic variation among the varieties, analysis of molecular variance (AMOVA) was performed with Arlequin v.3.5 (Excoffier and Lischer, 2010). Statistical significance for the data was assessed based upon 20,000 permutations. Again, this analysis was performed retaining only one individual per MLG found within each variety.

Nei *et al*. (1983) genetic distances (D_A_) were estimated among the varieties with MSA v.4.05 (Dieringer and Schlötterer, 2003), and a dendrogram was constructed using the Neighbor-Joining method (Saitou and Nei, 1987) implemented by PHYLIP v.3.6 (Felsenstein, 2005). Significance was estimated with 1,000 bootstrap replicates. The final tree was formatted with FigTree (http://tree.bio.ed.ac.uk/software/figtree/).

## Results

### Intra-varietal genetic diversity

The whole set of individuals (N = 390) presented two to eight alleles per locus and an average of 4.5 alleles over all loci (data not shown). All 14 varieties had higher observed heterozygosities than expected by random mating and the excess of heterozygotes was reflected in negative values of inbreeding (Table 2). Observed heterozygosities were also higher than expected heterozygosities for all soil types; the floodplain varieties had a slightly higher mean observed heterozygosity than did varieties from ADE and Oxisols, which had similar mean values of *H*_*O*_. The varieties *Tartaruga* and *Olho Roxo* from the floodplain, and *Aruari* and *Jabuti* from Oxisols, had private alleles. Most of the private alleles that were found across soil types had frequencies greater than 0.05 (Table S1).

**Table 2:** Genetic diversity parameters estimated for 14 bitter manioc varieties grown in three different soil types in Manicoré, Amazonas, Brazil, and for the set of varieties in each soil type, based on 10 microsatellite loci. Number of individuals (N), mean number of alleles (*Ā*), number of private alleles (Ap), number of multilocus genotypes (MLGs), number of unique multilocus genotypes (uMLGs), observed (*H*_*O*_) and expected (*H*_*E*_) heterozygosities and inbreeding coefficients (*f*). Soil types are coded as ADE (Amazonia dark earths), FP (floodplain) and OX (Oxisols). Significant values of *f* * (p < 0.05) are indicated.

Varieties/Soil types	N	*Ā*	Ap	MLGs	uMLGs	*H*_*O*_	*H*_*E*_	*f*
*Pirarucu Branco* ADE	20	1.5	-	2	1	0.495	0.250	-0.981^*^
*Pirarucu Branco* FP	20	1.6	-	2	1	0.505	0.255	-0.838^*^
*Pirarucu Amarelo* ADE	30	1.7	-	2	-	0.607	0.306	-0.862^*^
*Pirarucu Amarelo* FP	30	1.8	-	1	-	0.607	0.307	-0.754^*^
*Arroz* ADE	20	2.4	-	5	3	0.495	0.460	-0.076
*Arroz* OX	30	1.7	-	2	1	0.503	0.257	-0.752^*^
*Tartaruga* ADE	30	2.2	-	2	-	0.503	0.268	-0.530^*^
*Tartaruga* FP	30	2	3	5	3	0.707	0.361	-0.770^*^
*Tartaruga* OX	30	2.4	-	4	1	0.507	0.276	-0.445^*^
*Mãe Joana* FP	30	2.5	-	5	3	0.593	0.363	-0.451^*^
*Olho Roxo* FP	30	2	2	4	3	0.510	0.266	-0.695^*^
*Aruari* OX	30	2.4	3	3	2	0.583	0.329	-0.357^*^
*Jabuti* OX	30	2.6	1	6	3	0.567	0.445	-0.157^*^
*Roxinha* OX	30	1.5	-	1	-	0.500	0.250	-1.000^*^
ADE	100	3.3	1	9	4	0.532	0.505	-0.053^*^
Floodplain	140	3.6	6	17	10	0.590	0.510	-0.156^*^
Oxisols	150	3.6	5	15	7	0.531	0.526	-0.009

**Figure 2:**
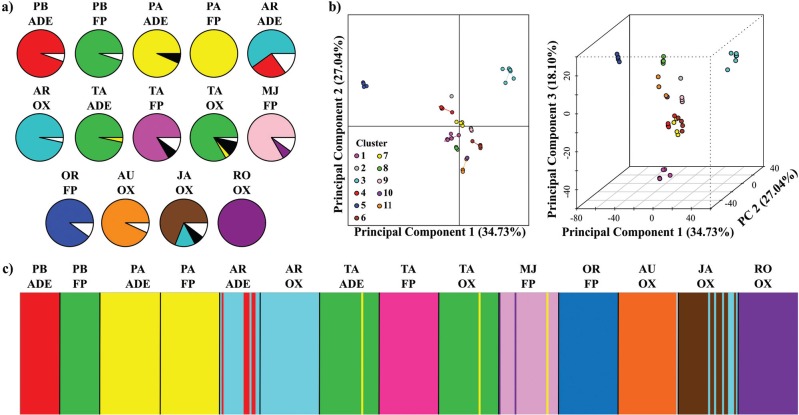
Comparison of genetic diversity and structure of bitter manioc varieties cultivated in three soil types in Manicoré, Amazonas, Brazil. a) Proportions of multilocus genotypes (MLGs), where different colors represent distinct MLGs. Black sections represent individuals that shared MLGs different from the dominant MLGs of their varieties (those in different varieties correspond to distinct MLGs). White sections represent unique MLGs for the whole set of varieties (and each section may be composed of more than one MLG). b) 2-D and 3-D results of DAPC analysis at *K*-means *=* 11 groups. c) Structure-like barplot based on individuals' membership probabilities to clusters generated in DAPC. Each individual is represented as a vertical line partitioned into colored segments, the length of which is the probability of the individual belonging to each *K*-means cluster. Figures were colored according to the preponderant MLG found in each variety. The identification of varieties follows the acronyms in Table 1.

A total of 35 MLGs were detected. Twenty-one of them occurred in only one individual in the whole set of individuals (unique MLGs, Table 2), and were distributed in eight varieties (Figure 2a). The other 14 MLGs were shared by two to 73 individuals (Table S2). High molecular genetic variability was observed among varieties, as shown by the multilocus genotypes (MLGs) detected within each variety (Figure 2a), confirming the polyclonal status of most varieties. *Pirarucu Amarelo* from the floodplain and *Roxinha* from Oxisols were the only two varieties with a single MLG in our sample. The other varieties showed at least two different MLGs, and the Oxisol variety *Jabuti* presented six different MLGs.

### Genetic structure among varieties

The *K*-means clustering method defined 11 clusters from the set of 14 bitter manioc varieties with DAPC (Figure 2b). Membership coefficients based on individuals' assignments to DAPC clusters were used to plot a Structure-like barplot (Figure 2c), which revealed that varieties were grouped in ten major clusters (one of the DAPC clusters was composed by only one individual of *Aruari* grown on an Oxisol). The number of DAPC clusters matched neither the number of varieties (14) nor the number of different vernacular names (9). Generally, varieties with the same vernacular name were in the same cluster, except for *Tartaruga* from the floodplain, which was in a different cluster from *Tartaruga* found in ADE and Oxisols. These latter two varieties were grouped in the same cluster as the variety *Pirarucu Branco* from the floodplain. Thus, from the total of 10 genetic groups recovered in DAPC, seven of them contained only one variety, another two were formed with varieties that had equal names but grown in different soils [*Pirarucu Amarelo* (from the floodplain and ADE), *Arroz* (from ADE and Oxisol)], and one was formed by varieties with different vernacular names [*Tartaruga* (from ADE and Oxisols) plus *Pirarucu Branco* (from the floodplain)]. DAPC and the comparisons of MLGs among varieties clearly showed that the same MLGs were present in different varieties. The varieties that were assigned to the same DAPC cluster showed the same MLGs, and the other varieties, each one assigned to a distinct cluster, had distinct predominant MLGs. These DAPC results were exactly the same as the DAPC based on the a-score criterion, and also highly congruent with the results of the Bayesian model-based analysis carried out in TESS (Figure S1).

Measures of genetic differentiation estimated among the set of different MLGs present in each bitter manioc variety generally revealed very high values for pairwise *F*_*ST*_ between different varieties (Table 3). Some varieties with equivalent vernacular names but grown in different soil types (*Tartaruga* on Oxisols and *Tartaruga* in the floodplain, for example) also presented high differentiation values.

**Table 3:** Genetic differentiation estimates (*F*_*ST*_) among 14 bitter manioc varieties grown in three different soil types in Manicoré, Amazonas, Brazil, using 10 microsatellite loci. Acronyms of the varieties are presented in Table 1. Values in bold typeface indicate comparisons between varieties with the same name, but grown in different soil types.

	PB ADE	PB FP	PA ADE	PA FP	AR ADE	AR OX	TA ADE	TA FP	TA OX	MJ FP	OR FP	AU OX	JA OX	RO OX
PB ADE														
PB FP	**0.472**													
PA ADE	0.136	0.259												
PA FP	0.027	0.154	**-0.568**											
AR ADE	0.167	0.229	0.163	0.055										
AR OX	0.494	0.319	0.370	0.297	**-0.040**									
TA ADE	0.209	-0.128	-0.124	-0.354	0.110	0.248								
TA FP	0.387	0.243	0.204	0.118	0.248	0.335	**0.120**							
TA OX	0.343	-0.157	0.125	-0.002	0.191	0.283	**-0.173**	**0.185**						
MJ FP	0.216	0.232	0.042	-0.097	0.202	0.338	0.031	0.247	0.149					
OR FP	0.324	0.393	0.091	-0.016	0.296	0.463	0.171	0.305	0.299	0.161				
AU OX	0.322	0.150	0.154	0.027	0.220	0.321	0.032	0.295	0.117	0.079	0.246			
JA OX	0.226	0.172	0.088	-0.017	-0.029	0.014	0.036	0.207	0.122	0.163	0.267	0.188		
RO OX	0.249	0.061	-0.039	-0.294	0.143	0.356	-0.226	0.331	-0.051	-0.024	0.242	-0.056	0.083	

When comparing the MLGs for each variety, the analysis of molecular variance (AMOVA) revealed that most genetic variation is found within varieties (81.38%), but with a large proportion of variation (Φ_*ST*_ = 0.186, significant at p < 0.001) also found among them (Table 4). Additional AMOVAs showed that little variation was found among soil types and among communities.

**Table 4:** Analysis of molecular variance for hierarchical groupings of 14 bitter manioc varieties grown in ADE, Oxisols and floodplain soils in communities in Manicoré, Amazonas, Brazil. The levels analyzed were: among the varieties; among varieties within soil types; among varieties within communities. d.f. = degrees of freedom.

Source of variation	d.f.	Sum of squares	Components of variation	Percentage of variation
Among varieties	13	74.24	0.54	18.62
Within varieties	74	175.41	2.37	81.38
Total	87	249.65	2.91	
Among soil types	2	15.61	0.05	1.84
Among varieties within soil types	11	58.63	0.50	17.20
Within varieties	74	175.41	2.37	80.97
Total	87	249.65	2.92	
Among communities	4	31.30	0.12	4.24
Among varieties within communities	9	42.94	0.44	14.95
Within varieties	74	175.41	2.37	80.81
Total	87	249.65	2.93	

**Figure 3:**
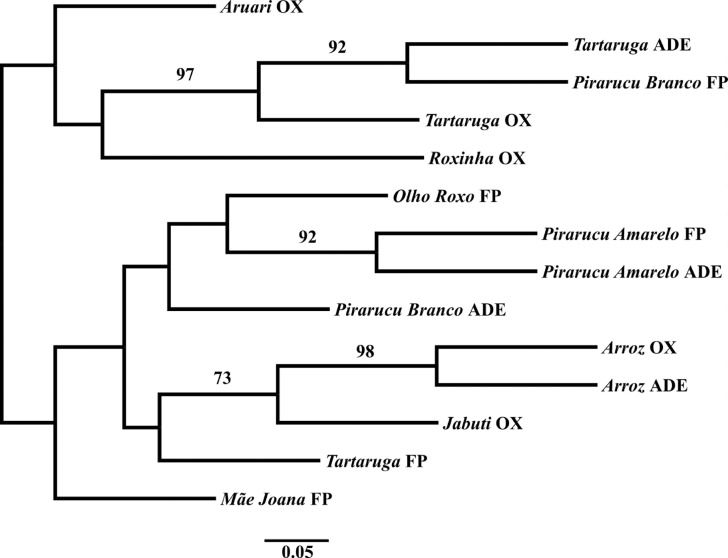
Neighbor-Joining dendrogram based on the Nei *et al*. (1983) genetic distance (D_A_), showing the relationships among 14 varieties grown in different soil types in Manicoré, Amazonas, Brazil. Soil types are coded as ADE (Amazonian dark earths), FP (floodplain) and OX (Oxisols). Bootstrap values greater than 70% are indicated.

Relationships among the varieties based on the genetic distance D_A_ (Nei *et al*., 1983) using the Neighbor-Joining algorithm corroborated the interpretations of the DAPC results (Figure 3). Varieties that were grouped in the same DAPC cluster were also closer to each other in the dendrogram, and had high bootstrap support. Reasonable bootstrap support was also found between *Jabuti* and *Arroz*. Soil type did not influence grouping of varieties.

## Discussion

### Maintenance and increase of genetic diversity within bitter manioc varieties

Considerable excess of heterozygotes was found for all bitter manioc varieties analyzed. Observed heterozygosities much higher than expected heterozygosities were also found in the manioc varieties cultivated by Palikur Amerindians in French Guiana studied by Pujol *et al*. (2005), because the Palikur frequently retain heterozygous volunteer seedlings in their swiddens. Pujol *et al*. (2005) and Pujol and McKey (2006) demonstrated that the size of seedlings was correlated with their multilocus heterozygosities and with their survival. During the weeding of swiddens, small seedlings are removed and larger ones are retained, and these more heterozygous seedlings become candidates for subsequent clonal propagation. Such a process allows the increase of genetic diversity (through incorporation of seedlings) and the maintenance of heterozygosity (through clonal propagation) in manioc varieties (Pujol *et al*., 2005).

The analysis of multilocus genotypes (MLGs) revealed that almost all bitter manioc varieties were polyclonal, with a preponderance of one clone and a mixture of other different genotypes, as observed by previous authors studying other manioc varieties in different parts of Amazonia (Elias *et al*., 2001; Peroni *et al*., 2007; Pujol *et al*., 2007). The incorporation of new MLGs in a given variety may occur by the incorporation of volunteer seedlings, and by unintentional admixture of plants from different varieties by farmers. Subsequent clonal propagation in the next cultivation cycle is responsible for the increase in the number of individuals with the new MLG within the variety. McKey *et al*. (2010b) comment on the contribution of somatic mutations to increase genetic diversity within clonally propagated crops. However, this is unlikely to explain the variation of MLGs found in this study, since the set of microsatellites used only covers a very small fraction of the estimated 772 Mb manioc genome (Awoleye *et al*., 1994; International Cassava Genetic Map (ICGMC), 2014).

Interestingly, about one-third of the farmers from two communities along the middle Madeira River intentionally use volunteer seedlings for clonal propagation, selecting the more attractive or healthy plants, and cultivating them separately for later evaluation (Fraser and Clement, 2008). These authors also observed farmers who unintentionally incorporated seedlings into existing varieties, allowing them to grow and later propagating them clonally without discriminating them from the other individuals of the cultivated variety. Extending their previous findings, Fraser *et al*. (2012) report that the proportion of farmers in six communities along the middle Madeira River that intentionally or unintentionally incorporate seedlings varies from 11%-32% and from 9%-33% per community, respectively. Hence, the incorporation of seedlings followed by clonal propagation plays an important role in the increase of genetic diversity in varieties cultivated along the middle Madeira River, just as occurs among the Makushi and Palikur Amerindians (Elias *et al*., 2001; Pujol *et al*., 2005, 2007).

### Genetic structure of bitter manioc varieties

Since pairwise *F*_*ST*_s were estimated with only one individual per MLG found within each variety, their values must be interpreted with caution. In general, varieties had high values of pairwise *F*_*ST*_s, which shows that the set of MLGs present in each variety is highly differentiated from that in other varieties. Low values of *F*_*ST*_ were found between varieties that were in different DAPC clusters and that were distantly related in the dendrogram, so these may be an artefact of the sample size reduction we used before calculating the estimates. However, it is clear that the set of MLGs in these varieties had substantially different allelic compositions (Figure 2a).

Fraser and Clement (2008) and Fraser (2010) observed similar agronomic traits in varieties from ADE and from the floodplain, and suggested that the varieties of these two soil types would be genetically closer and more divergent from the varieties grown in Oxisols. However, the individuals of *Tartaruga* and *Pirarucu Branco* from the floodplain did not cluster together with the individuals of the same variety from ADE (Figure 3). Additionally, the individuals of *Tartaruga* and *Arroz* from ADE were grouped with the individuals of corresponding varieties from Oxisols. There seems to be a tendency for varieties from the floodplain to be genetically differentiated from corresponding varieties in upland soils, *e.g*., *Tartaruga*. This may not be a general tendency, since individuals of *Pirarucu Amarelo* from the floodplain and ADE shared the same predominant MLG. Therefore, our results do not reflect the expectations of the hypothesis derived from ethnobotanical observations (Fraser and Clement, 2008; Fraser, 2010), because varieties from ADE and the floodplain were strongly differentiated, and because varieties clustered independently from soil types. The exception (*Pirarucu Amarelo* varieties from the floodplain and ADE) may be due to the fact that these were the only two varieties with the same vernacular name sampled in different soils of the same community. As observed by Fraser *et al*. (2012), the composition of bitter manioc varieties is clearly different on different types of soil in the middle Madeira River region, with higher differentiation between varieties from floodplain in relation to varieties from other types of soil. Genetic differentiation in bitter manioc varieties across soil types was also observed with molecular markers (Alves-Pereira *et al*., 2011). Farmers' convergent selection for similar ecological adaptations to nutrient-rich soils may be the reason why ADE and floodplain varieties share agronomic characteristics (Fraser *et al*., 2012), rather than shared phylogeny. Fast maturing and low starch varieties may be the outcomes of selection for rapid growth required in more intensive cultivation systems generally found in floodplain and ADE soils, as well as different soil conditions that may cause differences in stress to plants (Fraser *et al*., 2012). Among these stress conditions, the differences in annual flood regimes among different zones within the floodplain may influence the selection of an increasing number of varieties that may be suitable for cultivation there. This, in turn, may contribute to increasing genetic diversity within floodplain varieties, as well as increasing genetic differentiation of these varieties in relation to the varieties grown in other soil types. Therefore, the convergent selection proposed by Fraser *et al*. (2012) may be contributing to the maintenance and amplification of genetic diversity among bitter manioc varieties cultivated along the middle Madeira River.

The genetic structure revealed by DAPC and Bayesian analysis using TESS was corroborated by the relationships among varieties shown by the Neighbor-Joining dendrogram. The lack of bootstrap support for most of the branches may be due to the fact that, as shown by values of *F*_*ST*_ and detected by clustering in DAPC and TESS, the varieties sampled are so distinct from each other that it may be difficult to establish genetic relationships among them without many more microsatellite loci. The reasonable support found for the relationship between *Arroz* and *Jabuti*, also detected by DAPC and TESS, may be explained by the large number of MLGs shared by these two varieties. On the other hand, the clustering produced by DAPC and TESS showed that several individuals of *Arroz* from ADE were clustered with individuals of *Pirarucu Branco* from ADE, although they were not closely related in the Neighbor-joining dendrogram (Figure 3). This may be due to the higher similarity in allele frequencies, or even in allele composition, between the varieties *Arroz* and *Jabuti*, as suggested by the lower *F*_*ST*_ found between *Arroz* (ADE)/*Jabuti* (Oxisol) than between *Arroz* (ADE)/*Pirarucu Branco* (Floodplain) (-0.029 and 0.229, respectively), and by the composition of MLGs in these varieties.

The high levels of heterozygosity certainly reflected the high proportion of genetic variation within varieties revealed by AMOVA. On the other hand, the low genetic variation among manioc varieties in different soils and among communities was rather surprising since the floodplain communities had a generally different set of varieties when compared to the upland communities (ADE and Oxisols) (Alves-Pereira *et al*., 2011; Fraser *et al*., 2012). The fact that varieties with the same vernacular names, but sampled in different soils of different communities, were in general grouped in the same cluster of DAPC and TESS, reinforces the finding that the greatest genetic divergence is found among the varieties cultivated in the middle Madeira River, rather than among soils, and AMOVA results are in accordance.

DAPC cluster number 2 was represented by a single individual of the *Aruari* variety from an Oxisol. This individual presented one of the two unique MLGs found in *Aruari*. Given the general genetic distinction among varieties, it is possible that this individual belongs to another variety that has phenotypic similarities with *Aruari*, which might have caused the farmer to incorporate it in *Aruari*. It is also possible that this individual originated from sexual reproduction among varieties. Another intriguing result was that the variety *Pirarucu Branco* from the floodplain was shown to be almost identical to the variety *Tartaruga* from ADE and Oxisol. These varieties had the same predominant MLG, even though *Pirarucu Branco* was collected in a community different from the others. It is possible that the set of microsatellite loci were not sufficient for finding allelic differences between these varieties. Additionally, it is possible that genotype x environment (GxE) interactions may have an important role in explaining this finding, since phenotypic plasticity may be present among the traits that were most favored by selection during crop domestication (McKey *et al*., 2012). Boster (1985) discusses the “selection for perceptual distinctiveness”, in which selection is performed by farmers in order to recognize their varieties as a group of individuals that share a specific set of morphological characteristics that are different from other varieties. Therefore, it is possible that when the variety *Tartaruga* was moved from the uplands (ADE or Oxixols) to the floodplain (or vice versa) it underwent a name change due to differential phenotypic expression of the same genotype in different environments. There also may be a certain range of variation in the morphological characteristics that identify each variety (Boster, 1985; Elias *et al*., 2000; Duputié *et al*., 2009). In spite of having distinct MLGs, different individuals may present a set of morphological characteristics that are sufficiently similar to be grouped into the same range of phenotypic variation, which encourages farmers to give them the same varietal name. This is a possible explanation for the fact that the individuals of *Pirarucu Branco* from floodplain were genetically differentiated from those of ADE, and the same explanation may hold for the individuals of *Tartaruga* from floodplain, which were genetically distinct from the individuals of upland soils (ADE and Oxisol). GxE interactions were already documented for cyanogenic potential and nutrient composition in manioc (Bokanga *et al*., 1994; Burns *et al*., 2012), so it is not surprising to find other traits that may be due to GxE interactions. Studies on the variation of morphological characteristics among varieties of manioc grown along the middle Madeira river may be valuable to cast light on the occurrence of GxE interactions, and also on the genetic evidence found in our study.

Individuals that were clustered by DAPC and TESS separately from the rest of the individuals of a given variety displayed MLGs identical to predominant MLGs of a different variety (for example, between *Arroz* and *Pirarucu Branco* from ADE). These results may be due to farmers' confusion when assigning individuals to varieties. However, the term ‘confusion’ is relative because GxE interactions may influence the naming of varieties (Emperaire *et al*., 1998), and because the criteria for distinguishing varieties may vary among farmers (Salick *et al*., 1997; Sambatti *et al*., 2001; Emperaire and Peroni, 2007; Heckler and Zent, 2008).

Indeed, all the factors discussed here may be shaping the extent of genetic diversity and structure among bitter manioc varieties in the region of the middle Madeira River. These include, for example, the exchange of material among farmers, rates of incorporation of seedlings, and the naming of varieties may vary greatly among and even within different regions (Sambatti *et al*., 2001; Peroni and Hanazaki, 2002; Fraser and Clement, 2008; Oliveira, 2008). However, whatever the most important processes for the results reported in this study, they ultimately contribute to the maintenance of high genetic diversity within the varieties traditionally cultivated in communities of smallholder farmers along the middle Madeira River.

This study was the first to report on the genetic diversity and the genetic structure of bitter manioc varieties traditionally grown in different soil types in a small geographic region. Microsatellite variation revealed that traditional farmers of different communities along the middle Madeira River manage high levels of genetic diversity within some of the most frequently cultivated varieties in the region. Although there is large intra-varietal genetic diversity, varieties have distinct genetic features that, in general, differentiate one from the other. The genetic structure is primarily related to the varieties *per se*, and there was no clear tendency to greater similarity between varieties from the floodplain and ADE. Rather, there seems to be a differentiation between varieties grown in the floodplain and the same varieties grown in upland soils (ADE and Oxisols), so the hypothesis proposed by Fraser and Clement (2008) and Fraser (2010) is not supported by our study. It is more likely that farmers in the middle Madeira River region select their ADE and floodplain varieties for convergent adaptive traits (fast maturing, low starch), which are possibly associated with the similar ecological adaptations to fertile soils and short periods of growth (Fraser *et al*., 2012). The genetic differentiation of some of the floodplain varieties in relation to upland ADE and Oxisol varieties may be due to the selection of a distinct set of varieties for different flood regimes in different zones within floodplain soils. These may be a signature of physiological adaptations to flood regimes or edaphic properties, for example. The evaluation of morphometric characters in bitter manioc varieties grown in different soil types in the middle Madeira may be useful to better understand the possible existence of convergent adaptations.

Our study suggests that many factors may contribute to the maintenance and amplification of genetic diversity within bitter manioc varieties cultivated in communities along the middle Madeira River. We believe that it is worthwhile to invest in social policies that value the practices of smallholders, as they manage high levels of genetic diversity within their cultivated varieties of bitter manioc. These farmers must be seen as collaborators for the on-farm *in-situ* conservation of the genetic resources of manioc.

## Supplementary Material

The following material is available for this article:

Table S1 - Table of private alleles (with frequencies > 0.05) found in bitter manioc varieties in different soil types in Manicoré, Amazonas, Brazil.

Table S2 - List of multilocus genotypes (MLGs) across bitter manioc varieties in different soil types in Manicoré, Amazonas, Brazil.

Figure S1 - Criteria for choosing the optimal number of clusters (*K*) for TESS and DAPC, and comparison of individuals' assignments obtained with DAPC optimized with a-score and TESS.
