# Supplementary material for: High genetic diversity among and within bitter manioc varieties cultivated in different soil types in Central Amazonia
Source: Genet Mol Biol. 2017 Apr 10;40(2):468–79. doi: 10.1590/1678-4685-GMB-2016-0046 (PMC5488453; doi:10.1590/1678-4685-GMB-2016-0046)
Supplement: Supplementary file 1 [file 1415-4757-gmb-1678-4685-GMB-2016-0046-Suppl03.pdf]

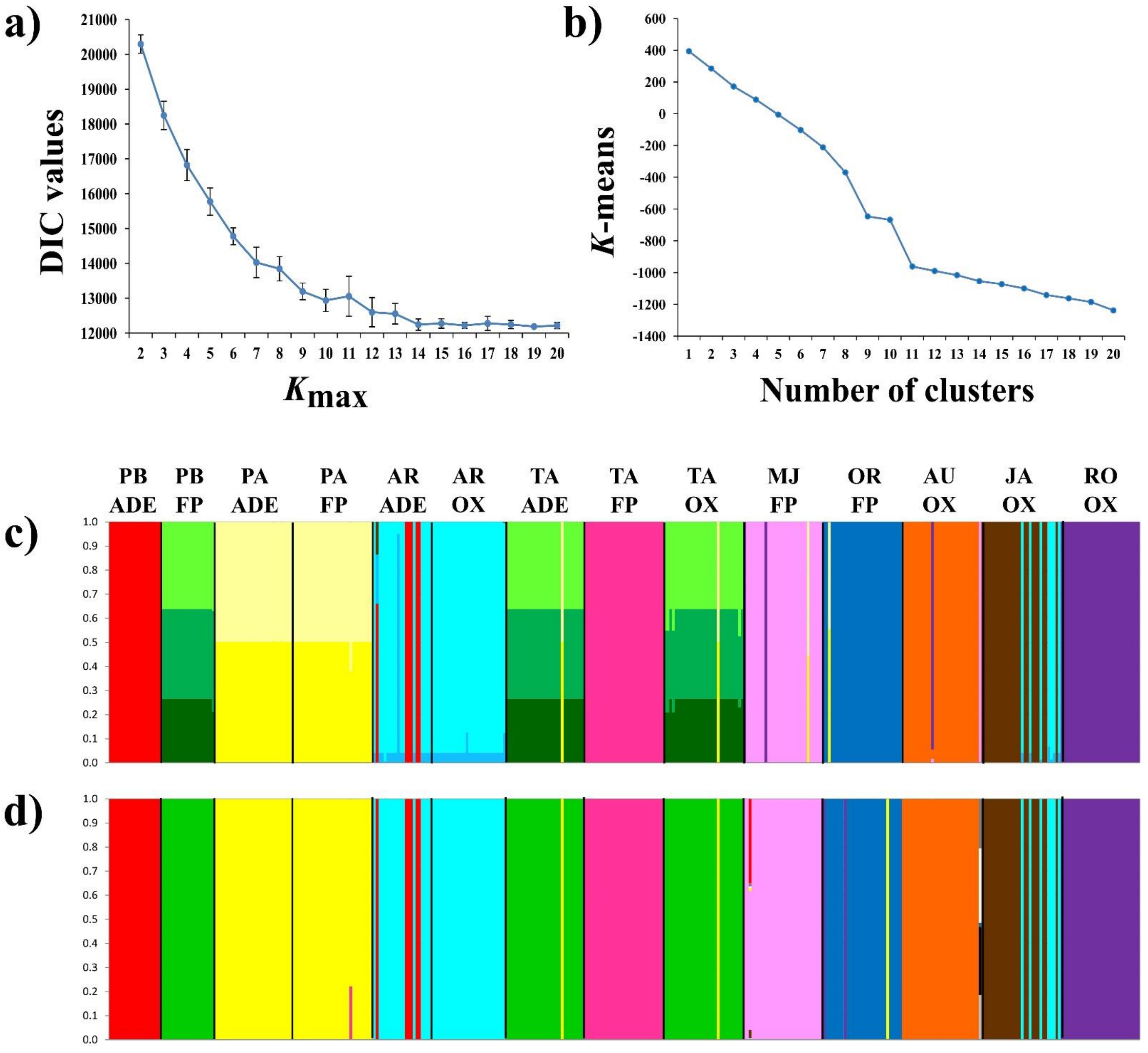

Figure S1 - Criteria for choosing the optimal number of clusters (*K*) for TESS and DAPC, and comparison of individuals' assignments obtained with DAPC optimized with a-score and TESS. a) Deviance information criterion (DIC) averaged over ten simulations (vertical black lines represent standard deviations) of K_{max} varying from 2 to 20 was used to select K_{max} = 14 in TESS. b) *K*-means value across the number of clusters (varying from 1 to 20) indicate that DAPC may be performed with 11 *a priori* clusters. c) Although DIC values indicated K_{max} = 14, the number of general groups obtained with TESS was 10 (the other four clusters are represented in the last individual of the variety *Aruari* grown on Oxisols). Acronyms of the varieties are presented in Table 1 of the main text. d) Individuals' assignments obtained for the DAPC optimized with a-score was performed retaining five principal components.
