# Supplementary material for: High genetic diversity among and within bitter manioc varieties cultivated in different soil types in Central Amazonia
Source: Genet Mol Biol. 2017 Apr 10;40(2):468–79. doi: 10.1590/1678-4685-GMB-2016-0046 (PMC5488453; doi:10.1590/1678-4685-GMB-2016-0046)
Supplement: Supplementary file 2 [file 1415-4757-gmb-1678-4685-GMB-2016-0046-Suppl01.pdf]

Table S1 - Table of private alleles (with frequencies > 0.05) found in bitter manioc varieties in different soil types in Manicoré, Amazonas, Brazil. Soil types are coded as ADE (Amazonian dark earths), FP (floodplain) and OX (Oxisols).

Varieties/Soil types	Locus	Allele	Frequency
Tartaruga FP	GA140	168	0.500
Tartaruga FP	SSRY89	118	0.500
Olho Roxo FP	GA126	188	0.500
Olho Roxo FP	SSRY13	212	0.483
Aruari OX	SSRY13	222	0.483
ADE	SSRY164	154	0.130
Floodplain	GA126	188	0.107
Floodplain	GA140	168	0.108
Floodplain	SSRY89	118	0.107
Floodplain	SSRY13	212	0.104
Floodplain	SSRY13	238	0.196
Oxisols	GA131	114	0.173
Oxisols	SSRY13	222	0.095
