# Supplementary material for: High genetic diversity among and within bitter manioc varieties cultivated in different soil types in Central Amazonia
Source: Genet Mol Biol. 2017 Apr 10;40(2):468–79. doi: 10.1590/1678-4685-GMB-2016-0046 (PMC5488453; doi:10.1590/1678-4685-GMB-2016-0046)
Supplement: Supplementary file 3 [file 1415-4757-gmb-1678-4685-GMB-2016-0046-Suppl02.pdf]

Table S2 - List of multilocus genotypes (MLGs) across bitter manioc varieties in different soil types in Manicoré, Amazonas, Brazil. For the detection of MLGs individuals with missing data were removed. N = number of individuals. Acronyms of the varieties are presented in Table 1 of the main text.

		**Shared MLGs**																**Unique MLGs**																		
**Varieties**	**N**	**11**	**4**	**25**	**5**	**1**	**8**	**20**	**30**	**17**	**34**	**10**	**19**	**26**	**35**	**2**	**3**	**6**	**7**	**9**	**12**	**13**	**14**	**15**	**16**	**18**	**21**	**22**	**23**	**24**	**27**	**28**	**29**	**31**	**32**	**33**
**PB ADE**	18									17								1																		
**PB FP**	20	19																		1																
**PA ADE**	30		28								1				2																					
**PA FP**	29		29																																	
**AR ADE**	20			12						5											1			1								1				
**AR OX**	29			28																										1						
**TA ADE**	30	29	1																																	
**TA FP**	30							25					2													1	1	1								
**TA OX**	30	25	1									3																	1							
**MJ FP**	30				2				25							1	1						1													
**OR FP**	30					27																1			1									1		
**AU OX**	29						27												1														1			
**JÁ OX**	29			4							20			2																	1				1	1
**RO OX**	30				30																															
**Total**	**384**	**73**	**59**	**44**	**32**	**27**	**27**	**25**	**25**	**22**	**21**	**3**	**2**	**2**	**2**	**1**	**1**	**1**	**1**	**1**	**1**	**1**	**1**	**1**	**1**	**1**	**1**	**1**	**1**	**1**	**1**	**1**	**1**	**1**	**1**	**1**
